# The future of data analysis is now: Integrating generative AI in neuroimaging methods development

**DOI:** 10.1162/imag_a_00241

**Published:** 2024-07-24

**Authors:** Elizabeth DuPre, Russell Alan Poldrack

**Affiliations:** Department of Psychology, Stanford University, Stanford, CA, United States

**Keywords:** computational science, open science, neuroimaging methods, artificial intelligence, code assistants

## Abstract

In this perspective, we highlight how emerging artificial intelligence tools are likely to impact the experiences of researchers conducting computational fMRI analyses. While calls for the automatization of statistical procedures date back at least to the inception of “data science” as a field, generative artificial intelligence offers new opportunities to advance field practice. We highlight how these tools are poised to impact both new neuroimaging methods development in areas such as image quality control and in day-to-day practice when generating analysis code. We argue that considering generative artificial intelligence as a catalyst for computational neuroscience—rather than as unique tools in their own right—can substantially improve its positioning in the research ecosystem. In particular, we argue that generative artificial intelligence will reinforce the importance of existing open science initiatives, rather than supplanting them. Overall, we call for clearer metrics by which neuroimaging results—whether generated by individual research teams or by generative artificial intelligence technologies—can be meaningfully compared.

## 1 Introduction

Alongside fields such as genomics and astronomy, neuroimaging has transformed over the last decade into a data intensive, “Big Data” field. With the emergence of funder-mandated data sharing (Jwa & Poldrack, 2022), individual researchers now have access to more neuroimaging data than ever before, with 406 terabytes accessed through the data sharing platform OpenNeuro from May 2020 to April 2021 alone (Markiewicz et al., 2021). While this growth has presented important opportunities to improve research practices (Gratton et al., 2022), it has also created unanticipated challenges. Researchers now need exponentially more training in data science and statistics than they did a decade ago. Many research supervisors do not have the necessary training to properly store, access, and analyze their data, and graduate degree programs lag behind evolving field standards. Open science communities such as Brainhack (Gau et al., 2021) as well as targeted educational initiatives such as NeuroHackademy (Huppenkothen et al., 2018) and NeuroMatch Academy (van Viegen et al., 2021) have stepped in to fill this gap with training materials and peer support networks. However, the scale of this challenge continues to outpace relatively slow changes both to training programs and to institutional hiring decisions, where relatively few roles focus on data science expertise.

In this space, the emergence of generative artificial intelligence (AI) has promised to revolutionize researchers’ experiences of the scientific process. Generative artificial intelligence (generative AI) is a class of algorithms that combine generative models with deep neural networks, allowing users to generate probable new text, images, and audio outputs from supplied human inputs such as text or image prompts. AI-assisted coding, in particular, has been argued to improve developer productivity—particularly in supporting relatively novice programmers (Peng et al., 2023; c.f.Orosz & Beck, 2023)—and has anecdotally been suggested to provide a more enjoyable coding experience by automating low-level details and reducing time to problem solution. Its potential to improve the scientific process is thus clear, though how it and other generative AI tooling are adapted by the broader community will ultimately shape its impact. There is thus a clear potential that it could be used to similarly accelerate methods development. While other reviews have considered emerging use cases of generative AI models for neuroimaging analyses (Gong et al., 2023), the potential impact of these methods on the scientific process itself remains underexplored. Here, we aim to directly consider these questions, overviewing the potential impact of AI-assisted tooling on neuroimaging field practice. We note that—while another important, related topic in this area—the influence of AI technologies on scientific writing and reviewing has ethical considerations for the current academic credit system (Nature, 2023), but we leave those for future discussion. Instead, we highlight how the emergence of generative AI methods is likely to impact both day-to-day practice and new analytic methods for neuroimaging. We further argue that these methods will play against the well-established open source ecosystem in neuroimaging, introducing new considerations for both developers and users of these tools.

## 2 A Historical Perspective: The Introduction of Data Science

While the mass adoption of generative AI technologies is a unique historical moment, we can use other methodological turning points to frame these developments. Perhaps the most relevant of these is the creation of “data science” as an approach to systematizing scientific data analysis. In 1962, John Tukey published “The Future of Data Analysis” in which he called for the creation of data science—a field contrasted with statistics in its unique focus on data analysis (Tukey, 1962).

Among other prescient arguments, Tukey emphasized the importance of developing automated, standardized approaches to as many statistical procedures as possible rather than relying on individual researcher expertise. Failure to do so, he suggested, would result in poor research practices as more data became available, since “most data analysis is going to be done by people who are not sophisticated data analysts and who have very limited time; if you do not provide them tools the data will be even less studied” (p. 22). Indeed, neuroimaging has found itself in exactly this position, as a lack of dedicated data science training siloed analytic expertise both within and across labs. The emergence of data standards such as BIDS (Gorgolewski et al., 2016) has allowed the Nipreps ecosystem (Esteban et al., 2019) to fill this gap in part by automating some common fMRI preprocessing workflows based on existing software platforms such as AFNI (Cox, 1996), FSL (Jenkinson et al., 2012), and ANTS (Tustison et al., 2021).

While extensions to other neuroimaging modalities are an obvious next step (though see, e.g., MNE-BIDS Pipeline for one existing example;Appelhoff et al., 2019), work across the Nipreps ecosystem has highlighted two core areas of work for neuroimaging methods development. First, the need to continue standardizing analysis practices in areas that have yet to develop a formal theory, such as image quality control. Second, the need to facilitate automation in the analysis of preprocessed fMRI datasets, where the exact analysis choices are specific to a particular research question and task design. Generative artificial intelligence (AI) may help to address both of these broad challenges.

Neuroscience occupies a relatively unique position among data-intensive fields in that AI promises to significantly impact both its methodological and theoretical foundations through, for example, “NeuroAI” (Richards et al., 2019;Zador et al., 2023). Even when considering only its methodological impacts, AI has the potential to cause significant harm—delaying rather than accelerating field progress—if researchers are ill-informed about the capabilities of these tools. We first consider how generative AI is likely to impact one specific aspect of neuroimaging data processing: quality control. We then consider how the more recent development of large-language models such as ChatGPT and GitHub Copilot^1^is beginning to change neuroimaging method development, and how these developments may impact the broader tooling ecosystem.

## 3 Using Generative AI for New Methods in Image Quality Control

While Tukey persuasively argued for automating existing statistical procedures, many neuroimaging methods do not have a clear path to automation. In particular, experimental procedures that do not have a clear metric of success are especially difficult to automate, as different scientists often prioritize different goals. One example is in experimental stimulus generation: while generative AI tools such as MidJourney and StableDiffusion can easily create a huge variety of images, individual researchers must choose which to prioritize in limited experimental time.

An even clearer example is in image quality control, where visual inspection remains the gold standard for the field. Researchers develop unique expertise in image quality control based on their training, largely in the context of specific scientific questions. It is not surprising, then, that quality control ratings differ even between experts (Taylor et al., 2023). Despite this uncertainty, the sheer amount of neuroimaging data to be checked has pushed researchers toward developing no-reference image quality metrics, with the idea that these can guide researcher inspection and downstream machine learning analyses (Bastiani et al., 2019;Esteban et al., 2017). This idea of using AI and machine learning techniques to improve quality control is not unique to neuroscience. As data cleaning comprises a significant portion of analyst time in industry settings, automated quality control for tabular data is an active area of research. While projects such as DirtyData (https://project.inria.fr/dirtydata/;Perez-Lebel et al., 2022) aim to provide theoretical guarantees in data cleaning, existing work points toward relatively nuanced—rather than robust—applications, in which the details of the problem drive performance (Cheng et al., 2022).

Despite this challenge, there is reason for optimism. In neuroimaging preprocessing, projects such as NoBrainer (Kaczmarzyk et al., 2023) and FastSurfer (Henschel et al., 2020) have used AI to dramatically reduce compute time for image-based tasks such as brain segmentation while maintaining high-quality outputs. There is, therefore, an obvious potential in using AI methods in neuroimaging data processing. Critically, however, these existing tools document their extensive validation against a large number of publicly available, labeled datasets. To date, large, labeled datasets for quality control validation are much harder to acquire.

One potential solution is to augment existing labels using crowd sourcing. Efforts such as Swipes for Science (Keshavan et al., 2019) and Brainmatch (Benhajali et al., 2020) have successfully generated quality control labels at scale using distributed, citizen science efforts. While these approaches have shown significant potential (Richie-Halford et al., 2022), the increasing availability of consumer-grade AI calls future work on crowdsourced platforms into question. That is, an increasing number of crowd workers are using tools such as ChatGPT to complete online crowd-sourced tasks, particularly those that allow for free-text input (e.g., to describe artifacts;Veselovsky et al., 2023). Training an algorithm on these AI-generated responses could lead to “model collapse,” in which performance significantly degrades over time (Shumailov et al., 2023).

More recently, efforts such as the Demonstrating Quality Control Procedures project (https://osf.io/qaesm/) have turned to focus on the task of label generation itself. The resulting quality control protocols may in turn be critical for future AI applications; indeed, generative AI could assist in this effort, by augmenting available data with a specific label (e.g., a “ringing artifact”) to assess inter-rater agreement. Certain quality control procedures and resulting labels, however, are likely to be specific to a given population. For example, lesion segmentation in stroke patients is an important image processing step that is not shared with normative population samples (Liew et al., 2018). In these cases, both limited data availability and strong privacy concerns are likely to impair extensive data labeling efforts.

Alternative learning paradigms such as federated learning provide one path forward, by allowing joint models to be learned via model sharing rather than data sharing (Stripelis et al., 2022). Similarly, neuroimaging foundation models (Caro et al., 2023;Thomas et al., 2022) may allow for transferring pretrained, data-intensive models to smaller, more specialized datasets. Importantly, however, both of these paradigms require a strong degree of data standardization to ensure that model features transfer appropriately between contexts, for example, experimental sites.

## 4 AI-Assisted Coding for Well-Defined Contexts

While Tukey encouraged the automatization of as many statistical procedures as possible, key neuroimaging analysis steps have resisted automation. This is in part because of the range of different analytic choices that a single dataset can support (Carp, 2012), making automation nontrivial. For example, two of the most widely used analyses of fMRI data are first- and second-level General Linear Modelling (GLM;Friston et al., 1995), which have served as cornerstones of fMRI analysis for over two decades. Only in the past few years, however, has dedicated tooling emerged to represent these very common analyses in a standardized format (e.g., FitLins;Markiewicz et al., 2022). Without a standardized and machine-readable input and output structure, automation through AI or other tooling is difficult.

As standardized GLM analyses are still being actively developed, it is no surprise that other, newer methods largely lack standardized formats. In these cases, it falls to the experimenter to ensure that their generated code is correctly executing the desired analysis. For researchers with no or minimal training in software engineering, this requirement can block access to certain scientific questions—or yield incorrect inferences (Soergel, 2014). In these cases, AI-assisted coding is an appealing solution to act as a “catalytic” research accelerator, lowering the activation energy of producing high-quality code. It does not provide a full free lunch, however. Instead, it shifts the burden from generating code to reviewing and testing code (Poldrack et al., 2023). For example, if AI-assisted coding is used to generate tests, does a failing test indicate a problem with the test or the tested code? Does the derived code produce sensible inputs and outputs? These questions still require active input from researchers who are comfortable with software engineering principles.

While this approach is tractable at some levels of analysis (e.g., to write a single function within a larger workflow), scaling it to a complete neuroimaging experiment exponentially increases the possibility of errors. Since there is rarely a single metric which researchers can benchmark their results against, it is also difficult to isolate when AI-generated code is producing meaningful variability or introducing subtle errors. Having access to data and code supporting previous results can provide a meaningful sanity check, serving as a higher-level test. That is, researchers can assess: With the assistance of GitHub Copilot or ChatGPT, can I generate a version of this existing code that runs on my data? If I re-run it on the original data, do I see similar results to the original analysis? This provides an index of how robust and replicable (The Turing Way Community, 2023) a previous result is, as well as a confirmation that new code is correctly reproducing the intended analyses. We provide an example workflow for generating new research code using these tools inBox 1. Critically, however, it relies on the availability of open data and code for existing experiments.

The importance of data and code sharing in this context points toward open science solutions. We thus turn next to the interaction of AI-driven science with the existing open source and open science landscapes.

Box 1 **Example workflow for code generation.**

Working with large language models can accelerate computational analyses. While many workflows are possible when working with these models to generate code, we provide one generic example here to help orient the reader.

1. Use the developer API to set a random seed value and track the system fingerprint, whenever possible, to assist in reproducing the system behavior.
2. Prompt the model with a specific query. Some suggestions^2^include:
  - Create Python code to generate new test data for the following function.”
  - Create a Python class to perform spatial smoothing on a NifTI image using a Gaussian filter.”
  - Given the following function, generate associated tests.”
  - Add docstrings to the included code.”
  - Refactor the provided code to make it easier to read.”
3. Vary the random seed and temperature to better understand the variability of behavior of the model across parameterizations and adapt suggested code accordingly.
4. Test that the model-suggested code runs on real data and generates sensible outputs, if applicable.

## 5 Open Data, Open Code, Open AI? How AI-Assisted Tooling Will Interact with Open Science

Open science has been a critical driver of methods development in neuroimaging over the last decade, yielding large-scale field standardization efforts for the most common preprocessing workflows (Kiar et al., 2023). Open science was not a specific focus of “The Future of Data Analysis” (Tukey, 1962); this reflects the change in the research landscape, however, rather than the potential effects of open science efforts. Indeed, in the years since “The Future of Data Analysis” was published, open science practices of data and code sharing have become a driving force both in neuroimaging and in many other data intensive fields (Donoho, 2017). As reviewed above, it is clear that many of these same open science practices interact with AI-assisted methods. In particular, AI-assisted coding will catalyze new research by lowering the barrier for coding, allowing researchers with little formal data science training to run otherwise inaccessible neuroimaging analyses. Additional generative AI technologies are likely to be useful in refining fMRI methods, such as neuroimaging quality control.

An obvious question is how AI-assisted tooling will change open science and its practice in neuroimaging methods development. That is, will AI-assisted tooling diminish open science practices such as data and code sharing, when data and code can simply be created on command? Evidence from other fields suggests the opposite. Recently, David Donoho—a leading figure in the field of data science—argued that the commercial success of AI reflects a robust data science culture in empirical machine learning (Donoho, 2023). He advocated for not only open code and data sharing, but also explicit metrics by which individual analyses can be compared, such as performance on a public prediction challenge. While prediction challenges have seen limited success in neuroimaging to date (Traut et al., 2022), the idea of clear metrics by which neuroimaging results can be compared will be critically important to more fully adopt generative AI technologies into neuroimaging methods development. Without clear outcome metrics, strong human-in-the-loop systems will be necessary to review individual AI applications, falling short of Tukey’s call for automated analysis. Greater standardization efforts are, therefore, a critical step in adopting generative AI technologies. Encouragingly, open science efforts such as the Demonstrating Quality Control Procedures project are beginning to chart a path toward standardization for previously ambiguous metrics, such as visual quality control. Nonetheless, significant work remains to be done, including making the results of neuroimaging analyses FAIR (Findable, Accessible, Interoperable, and Reusable;Poline et al., 2022) so that they can be objectively assessed by independent investigators. While generative AI technologies may help in this process (e.g., by developing new examples for human labeling), deeper progress will remain tied to human-led standardization efforts.

Focusing on the idea of automated analysis suggests that generative AI will not replace existing open science initiatives any time soon. Instead, it will require researchers to produce not only open data and open code, but also clear outcomes by which individual experiments can be compared. Initiatives such as the Neuroimaging Analysis Replication and Prediction Study (NARPS;Botvinik-Nezer et al., 2020) confirm that teams of researchers conducting the same experiments on the same neuroimaging data can produce substantial differences in results. This work does not provide, however, a clear framework to compare each team’s results. Multiverse analyses (Dafflon et al., 2022) or more general “vibration” analyses (Bhagwat et al., 2021) may be a useful mechanism to calibrate the range of possible results for a given experiment. Ideally, these calibrations could guide future work to generate open metrics. For the moment, however, continuing to openly share code and data to allow for these analyses—either within or across research teams—will be necessary to better situate emerging AI-assisted methods.

## 6 Conclusions

As a data intensive field, neuroimaging methods are increasingly reliant on data science practices to drive methodological innovation. Nonetheless, most researchers lack necessary data science training. Generative AI tooling may help to fill this gap; in doing so, however, it will strongly interact with existing systems for neuroimaging methods development. Many of these have matured in coordination with open science efforts, particularly those that focus on data and code sharing. In our view, the emergence of AI tooling will not supplant these efforts but instead reinforce their importance.

That is not to say that the interaction between open science and generative AI methods will be entirely synergistic. For example, defaced neuroimaging data are considered private data under The European Union’s General Data Protection Regulations (GDPR), and nonanonymized neuroimaging data are considered private by even more countries, such as the United States. Directly providing neuroimages to proprietary tools such as ChatGPT or GitHub Copilot, therefore, is broadly unethical. Other examples of potential AI and open science interactions are somewhat murkier. For example, AI-assisted coding could conceivably lower the barrier to collaboration with Research Software Engineers (RSEs;Woolston, 2022) or with the broader open source ecosystem of neuroimaging tools, such as NIPY (Brett et al., 2009). That is, by making it easier to produce code, researchers may be able to more easily contribute to open source tools, supporting more analytic choices than would otherwise be possible. Nonetheless, there are several potential pitfalls to this approach, especially given the already limited developer time for code review and maintenance in open source projects (Eghbal, 2016) and the limited institutional support for RSEs to date.

Overall, we suggest that best capitalizing on AI-assisted tooling will require returning to the core principles of data science. In particular, active work on clear metrics by which research results can be compared is likely to significantly improve both the adoption of AI-assisted tooling and the state of neuroimaging methods as a whole.

## Data Availability

No additional datasets or code are associated with this paper.

## Footnotes

1 Although we list specific models here for clarity, many of the larger language and image generation models are currently facing legal challenges for their use of associated training data. The reader should thus be alert to changes in the legal status of these models.

2 Adapted fromPoldrack et al. (2023).
